# Comparative evaluation of human breast milk, cow milk, infant formula and almond milk on childhood cariogenicity: An *in vivo* study

**DOI:** 10.6026/973206300221416

**Published:** 2026-03-31

**Authors:** Ashmita Debnath, Deepti Jawa, Shipra Jaidka, Nishi Chaudhary, R Selseya, Subhraneel Bhattacharya

**Affiliations:** 1 Department of Pediatric and Preventive Dentistry, Divya Jyoti College of Dental Science and Research, Modinagar, Uttar Pradesh 201204, India

**Keywords:** Early childhood caries (ECC), pediatric oral health, cariogenic potential, salivary pH, milk substitutes

## Abstract

Various feeding practices significantly influence pediatric oral health and cariogenic potential, necessitating evaluation of different milk types on key oral environmental markers. Hence, this *in vivo* study randomly divided 100 children (8 months-3 years) into four equal groups based on primary consumption of human breast milk, cow milk, infant formula or almond milk. Salivary pH, plaque pH and *Streptococcus mutans* colony-forming units were measured before and after feeding to assess cariogenic potential across groups. Human breast milk demonstrated superior cariostatic properties, maintaining highest pH levels and lowest bacterial counts compared to other milk types. These findings advance pediatric dentistry by confirming breast milk-s optimal role in minimizing early childhood caries risk and supporting preventive oral health strategies.

## Background:

Dental caries is one of the most common chronic infectious diseases worldwide and remains a major public health challenge in young children, who are particularly vulnerable to early childhood caries (ECC). ECC is a rapidly progressing form of decay that often begins soon after tooth eruption and is strongly associated with feeding behaviours that expose teeth to fermentable carbohydrates for prolonged periods, especially during night-time or between meals [1]. Beyond causing pain and infection, ECC can impair eating, speaking, sleep, growth, school performance and overall quality of life, while also increasing the risk of future caries in both primary and permanent dentitions [2]. The development of dental caries is multifactorial, involving complex interactions among dietary sugars, dental plaque biofilm, susceptible tooth surfaces, saliva and time. Within plaque biofilm, cariogenic bacteria such as *Streptococcus mutans* metabolise fermentable carbohydrates to organic acids, leading to a fall in plaque pH below the critical threshold for enamel demineralisation [3]. Repeated and prolonged episodes of low pH shift the balance towards net mineral loss and cavitation, especially in newly erupted primary teeth with thinner enamel. *S. mutans* is considered a key etiologic organism due to its strong adhesion to tooth surfaces, ability to synthesise extracellular polysaccharides, form acidogenic and aciduric biofilms and thrive in low pH environments [4]. Consequently, factors that influence its early colonisation and growth-particularly infant feeding practices-plays a crucial role in determining caries risk in early life. Feeding patterns during infancy and early childhood shape the oral microbial environment and can either protect against or predispose to ECC.

Human breast milk is widely regarded as the ideal nutrition for infants, providing balanced macronutrients along with immunoglobulins, antimicrobial peptides, oligosaccharides and a diverse microbiota that support host defence and may inhibit the proliferation and adhesion of cariogenic bacteria [5]. Its mineral content and bioactive components contribute to healthy oral and craniofacial development and controlled consumption patterns are generally considered compatible with caries prevention. However, maternal health issues, work constraints, sociocultural factors and perceived or actual low milk supply often necessitate the use of alternative feeds such as infant milk formula, cow milk and plant-based milks like almond milk [6]. These substitutes differ markedly in composition and thus in their potential cariogenic impact. Cow milk contains lactose as its primary carbohydrate but also provides casein, calcium and phosphate, which can enhance remineralisation and buffer plaque acids, thereby moderating caries risk under normal consumption patterns [7]. Infant milk formulas, although designed to approximate the nutritional profile of breast milk, frequently incorporate additional fermentable carbohydrates such as sucrose, glucose polymers or corn syrup solids, which may increase acid production by dental biofilm and lower plaque pH more dramatically [8]. Almond milk and other plant-based beverages, especially commercially sweetened varieties, often lack the protective phosphoproteins and high mineral content found in mammalian milk while containing added sugars that readily fuel acidogenic bacteria. Prolonged, frequent or nocturnal exposure to these sweetened liquids through bottles or sippy cups can significantly reduce plaque pH and favour early colonisation by *S. mutans* in very young children [9]. In the context of rising ECC prevalence and increasing use of milk substitutes driven by allergy, lactose intolerance, lifestyle choices and marketing, clarifying the relative cariogenicity of commonly used milk types is essential for preventive dentistry. Understanding how human breast milk, cow milk, infant formula and almond milk affect salivary pH, plaque pH and *S. mutans* levels in infants and toddlers can provide evidence- based guidance for parents, paediatricians and oral health professionals. Therefore, it is of interest to evaluate and compare these effects in children aged 8 months to 3 years, with the overarching goal of supporting informed feeding choices and strengthening early preventive strategies against ECC.

## Materials and Methods:

For this study, 100 children aged 8 months to 3 years were selected and randomly divided into four equal groups based on their primary milk intake: Group 1 - human breast milk, Group 2 - cow milk, Group 3 - infant milk formula and Group 4 - almond milk.

## Inclusion criteria:

[1] Children aged 8 months to 3 years.

[2] Children who do not have lactose intolerance.

[3] Children do not have any specific disease.

[4] Nursing lactating mothers willing to give their informed consent.

## Exclusion criteria:

[1] Children do not have any systemic illness

[2] Children with physical or intellectual disabilities are excluded

[3] Drugs affecting salivary flow are excluded.

[4] The Children those who are lactose intolerant.

Each child was scheduled in the morning and instructed to avoid any food or drink except water for at least 1 hour before examination to standardise baseline conditions. Salivary pH was recorded intraorally using pH indicator strips placed in the floor of the mouth; baseline readings were taken before milk ingestion and post-ingesion readings were recorded 45 minutes after consumption of the allocated milk. Plaque samples were collected from the labial surfaces of primary maxillary anterior teeth using a sterile spoon excavator and immediately transferred into tubes containing 1 ml of normal saline. The plaque suspension was used for two purposes: (1) plaque pH assessment using a calibrated digital pH meter and (2) microbiological analysis. The pH meter electrode was rinsed and dried between measurements to avoid cross-contamination and plaque pH was recorded at baseline and 45 minutes after ingestion of the test milk. For microbiological evaluation, aliquots of the plaque suspension were inoculated onto Mitis Salivarius Bacitracin agar plates using a standardised loop technique. The plates were incubated at 37°C for 48 hours in a microaerophilic environment, after which colonies exhibiting the characteristic morphology of *Streptococcus mutans* were identified and counted. Colony-forming units (CFU) were expressed as CFU/ml of plaque suspension. All procedures were performed by a single calibrated examiner to minimise inter-examiner variability and all equipment was standardised and calibrated before data collection. The recorded parameters for each child included salivary pH, plaque pH and *S. mutans* CFU at baseline and 45 minutes following ingestion of the respective milk type. Comparative analysis between the four groups was planned to assess changes in these oral parameters and to determine the relative cariogenic potential of human breast milk, cow milk, almond milk and infant milk formula.

## Results:

Human breast milk (Group 1) produced the highest increase in salivary pH(1.843±0.079), followed by cow milk (Group 2), infant milk formula (Group 4) and almond milk (Group 3). All groups showed a significant rise from pre- to post-intervention (p=0.001) (Table 1, Figure 1 - see PDF). Plaque pH also increased significantly in all groups(p=0.001), with the greatest rise in Group 1(2.508± 0.074), followed by Groups 2, 3 and 4 (Table 2, Figure 2 - see PDF). All milk types caused a significant increase in *S. mutans* counts(p=0.001). The percentage increase was lowest with human breast milk (23.65%) and highest with infant milk formula (47.97%), with cow milk and almond milk in between. Overall cariogenicity ranked: Infant milk formula > Almond milk > Cow milk > Human breast milk (Table 3, Figure 3 - see PDF).

## Discussion:

Early childhood caries (ECC) is strongly linked to feeding practices and the frequency and nature of fermentable carbohydrate exposure in the oral cavity, particularly in infants and toddlers. De Grauwe *et al.* (2004) [10] emphasised that ECC is a multifactorial disease in which diet, oral microbiota and host factors interact, with early colonisation by *Streptococcus mutans* playing a central role. Lemos *et al.* (2019) [11] further described *S. mutans* as a key cariogenic species because of its strong adhesion, biofilm formation and ability to thrive in acidic conditions, making it a primary target when evaluating the cariogenicity of infant feeding practices. The present study, which compared the effects of human breast milk, cow milk, infant milk formula and almond milk on salivary pH, plaque pH and *S. mutans* counts, aligns with earlier work investigating dietary substrates and plaque acidogenicity. Prabhakar *et al.* (2010) [12] assessed the acidogenicity of plain and sweetened cow-s milk and concluded that, in the absence of saliva, both cow-s milk and human breast milk can be cariogenic, underscoring the importance of the oral environment and exposure conditions. Pandey *et al.* (2022) [13] reported that various infant milk formulas significantly lowered plaque pH to levels comparable to or even greater than sucrose solutions, suggesting that their carbohydrate content confers substantial cariogenic potential. These findings support the present observation that infant formula produced more pronounced reductions in oral pH and higher *S. mutans* counts compared to human breast milk. Human breast milk has been widely studied for its complex balance of nutritional and protective factors. Avila *et al.* (2015) [14] and Branger *et al.* (2019) [15] found that, in general, breastfed children have a lower caries experience than bottle-fed children, although prolonged, on-demand nocturnal breastfeeding beyond 24 months may increase caries risk when combined with poor oral hygiene and high sugar intake. Breast milk contains immunoglobulins, antimicrobial peptides, oligosaccharides and a diverse "human milk microbiota" that may help modulate colonisation by *S. mutans* and support a more favourable oral ecology [15]. Notarbartolo *et al.* (2022) [16] described Firmicutes, Proteobacteria and Actinobacteria as part of the core human milk microbiome, while Kim and Yi (2020) [17] highlighted the role of bacterial extracellular vesicles in vertical microbial transmission, offering further biological plausibility for the relatively lower cariogenic profile of breast milk observed in this study. Cow milk, in contrast, presents a different balance of risk and protection [16]. While it contains lactose, it is also rich in casein, calcium and phosphate, which can enhance remineralisation and buffer plaque acids. Shah (2016) [18] reported that adding sugar to milk did not always drop the pH below the critical 4.5-5.5 range, but studies by Masih *et al.* (2010) [19] showed that sweetened milk significantly lowered plaque pH in children, underscoring that added sugars and feeding frequency are decisive factors. In the present study, cow milk showed intermediate behaviour-less cariogenic than infant formula and almond milk but not as favourable as breast milk-which is consistent with the literature suggesting a conditional protective effect when unsweetened and consumed appropriately. Plant-based alternatives, particularly almond milk, have gained popularity but pose specific concerns for dental health. Lee *et al.* (2018) [9] assessed the cariogenic potential of various almond and soy milks and found that sucrose- sweetened almond milk produced the largest amount of biofilm growth and acid production compared to whole cow milk, recommending that clinicians warn patients about the cariogenic risk of sweetened almond milks. Much commercial almond milk marketed for children contain added sucrose or cane sugar and lack the phosphoproteins and high mineral content of mammalian milks, a combination that favours rapid acidogenesis and *S. mutans* proliferation [20]. The present study-s finding that almond milk caused greater drops in salivary and plaque pH and higher *S. mutans* counts than breast and cow milk reinforces these concerns. Saliva and its physicochemical properties form an essential part of the caries risk picture. Lower salivary pH, reduced buffering capacity and altered flow rate are associated with higher caries activity, whereas better hydration, higher pH and stronger buffering capacity are protective [21]. The current study, by simultaneously assessing salivary and plaque pH alongside microbial counts, integrates substrate effects with host factors and shows that breast milk best preserved a more neutral pH while limiting *S. mutans* proliferation compared to the other milk types.

## Conclusion:

Human breast milk showed the most favourable effect on oral health, maintaining higher salivary and plaque pH and the lowest *Streptococcus mutans* counts among all milk types. Cow milk exhibited moderate protective effects, while infant formula and almond milk led to greater pH drops and higher bacterial counts, suggesting higher cariogenic potential. These results affirm breast milk as the optimal primary feed for lowering early childhood caries risk and emphasise prudent formula or plant-based milk use with proper oral hygiene practices.
